# Dopamine precursor depletion affects performance and confidence judgements when events are timed from an explicit, but not an implicit onset

**DOI:** 10.1038/s41598-023-47843-w

**Published:** 2023-12-11

**Authors:** Ljubica Jovanovic, Morgane Chassignolle, Catherine Schmidt-Mutter, Guillaume Behr, Jennifer T. Coull, Anne Giersch

**Affiliations:** 1 Inserm 1114, Centre for Psychiatry, University Hospital of Strasbourg, Strasbourg University, Strasbourg, France; 2 Laboratoire des Systèmes Perceptifs, École Normale Supérieure, PSL University & CNRS, Paris, France; 3 Laboratoire des Neurosciences Cognitives (LNC), Aix-Marseille University & CNRS, Marseille, France; 4 CIC Inserm 1434, CHU Strasbourg, Strasbourg, France

**Keywords:** Human behaviour, Perception

## Abstract

Dopamine affects processing of temporal information, but most previous work has tested its role in prospective tasks, where participants know in advance when the event to be timed starts. However, we are often exposed to events whose onset we do not know in advance. We can evaluate their duration after they have elapsed, but mechanisms underlying this ability are still elusive. Here we contrasted effects of acute phenylalanine and tyrosine depletion (APTD) on both forms of timing in healthy volunteers, in a within-subject, placebo-controlled design. Participants were presented with a disc moving around a circular path and asked to reproduce the duration of one full revolution and to judge their confidence in performance. The onset of the revolution was either known in advance (explicit onset) or revealed only at the end of the trial (implicit onset). We found that APTD shortened reproduced durations in the explicit onset task but had no effect on temporal performance in the implicit onset task. This dissociation is corroborated by effects of APTD on confidence judgements in the explicit task only. Our findings suggest that dopamine has a specific role in prospective encoding of temporal intervals, rather than the processing of temporal information in general.

## Introduction

Dopamine has long been implicated in the ability of humans and other animals to encode, compare and produce temporal intervals. Pharmacological studies in animals^1–4^, as well as direct manipulation of dopamine signalling^5^, suggest that dopamine affects behaviour in various temporal tasks. In humans, the administration of dopaminergic antagonists impairs the discrimination of temporal intervals^6–8^, and biases performance in (re)production of recently presented temporal intervals^9–12^, although this relationship might be more intricate than in rodents^9,10,12^.

It has been proposed that the ability to time temporal intervals in a prospective manner (the beginning and the end of the interval to be evaluated is clearly signalled to participants) is subserved by a stopwatch-like, i.e. clock mechanism, which is reset at the onset of an event to-be-timed. The elapsed time is then measured by accumulation and counting of “ticks” of the clock (^13–16^, although see^17–19^). However, the ability to estimate temporal intervals is not limited to prospective duration estimation. We are able to estimate durations of events in the past, even without explicitly timing them, and this mechanism has been termed *retrospective* timing^20–23^. In fact, in our environment we are often exposed to events whose onset and duration are not known in advance, but we are still able to evaluate their duration after the events have elapsed. Without an explicit onset, the stopwatch-like mechanism cannot be used. However, evidence for dissociations between the representations and mechanisms of prospective and retrospective timing is scarce^17,24^ and it is not clear to what extent mechanisms subserving retrospective temporal estimation rely on dopamine signalling.

In the work presented here, we used the acute phenylalanine and tyrosine depletion method (APTD,^25,26^) to decrease dopamine synthesis, which has previously been shown to decrease temporal estimation accuracy in both perceptual^6^ and motor^9,12^ timing tasks. We asked participants to reproduce the duration of the revolution of a disc that moved around a circular path at varying speeds, and contrasted performance in two temporal reproduction tasks. In the *explicit onset* task, the disc made one full revolution and participants knew in advance when to start and end timing its duration (as in a classical prospective timing task). In the *implicit onset* task, the disc made more than one full revolution, and participants were asked to reproduce the duration of the final revolution only. Crucially, participants did not know in advance when the stimulus would stop. In other words, there was no information regarding when the event to be timed would *start* until the disc had stopped and the event had elapsed^27^. We hypothesised that if dopamine affects duration processing in general, dopamine precursor depletion should affect both explicit and implicit onset tasks similarly. In contrast, if its effects are specific to mechanisms underlying processing of durations with explicitly cued onsets, such as the use of the “internal clock”, the results may differ in the two tasks. In order to test the hypothesis that dopamine affects the uncertainty of temporal estimates^28–30^, we also asked participants to evaluate their confidence that their own decisions were correct on each trial^31–33^. Previous work has implicated dopamine in the metacognitive evaluation, suggesting that APTD may affect temporal metacognition independently of its effect on temporal processing^34–37^. Confidence judgements might also be indirectly affected by APTD, via increased uncertainty in temporal estimates. In this case we would expect APTD effects on confidence judgements to vary as a function of its effects on performance in the timing tasks.

## Methods

### Participants

Twenty-six healthy volunteers (17 women, mean age = 25.5 years), were recruited. The study was approved by the French Ethics Committee CPP Nord-Ouest I (CPP0013/2017) and registered with Agence Nationale de sécurité du médicament et des produits de santé (2016-A01598-43), and participants gave written informed consent. Participants underwent an assessment by a psychiatrist, were screened for neurological, psychiatric disorders (Beck Depression Inventory (BDI) and Community Assessment of Psychic Experiences, CAPE-42) and substance abuse. They all had normal or corrected-to-normal vision. All methods were performed in accordance with the relevant guidelines and regulations. Four participants abandoned the study before the first session (vomiting caused by drink ingestion which is a known side-effect (e.g.^38^) or distress during blood sampling (one participant)), and three participants completed only the first session. In total, six participants could not complete the protocol because of vomiting: three after the placebo and three after the APTD drink.

In total, 19 participants completed both sessions (11 women, mean age = 26.26). The sample size is similar to previously published studies on time perception of healthy volunteers manipulating dopaminergic signalling (e.g.^6,9,10,28^). Participants also completed several other temporal estimation tasks, reported elsewhere^12^, as well as Visual Analogue Scales reporting subjective levels of arousal, fatigue and mood before and after each session (see Supplementary material for details). Data from two participants were not included in the final analyses because they gave random responses across different tasks (no correlation between presented and reproduced duration).

### APTD manipulation

Participants were tested in a within-subject, double-blind, placebo-controlled, counterbalanced design. Each individual participated in two sessions: in one session they ingested a nutritionally balanced (BAL) amino acid drink, and in the other session, a drink lacking the dopamine precursors phenylalanine and tyrosine (APTD,^25,26,39,40^). Among the participants whose data was included in the analyses, eight had APTD drink in the first session (five of which completed the implicit condition first) and nine had BAL drink (four of which completed the implicit condition first). Since nausea and vomiting caused by the drink was not happening exclusively in APTD sessions it could not be used as a clue that would undermine the double-blinding process. Details on drink composition and mode of administration are reported in Supplementary material.

To assess plasma amino acid levels, blood samples were taken twice during each of the two testing sessions: prior to ingestion of the drinks and approximately 4 h post-ingestion, just before the beginning of the behavioural tasks. Blood samples were centrifuged immediately (24 °C, 3000 rpm, 10 min) and plasma was stored at − 80 °C until analysis.

The day prior to the experimental session, participants followed a low-protein diet provided by the investigators, were asked to abstain from alcohol, take a maximum of three caffeinated drinks and fast from midnight (except for water). All participants started experimental sessions in the morning (8 h or 9.30 h) and the behavioural tests approximately 4 h later (12 h or 13.30 h). Before each session, the absence of pregnancy was verified. The temporal interval between the two sessions was a minimum of 1 week (mean interval = 10.26 days, range = 7–22 days). The study was conducted in the Clinical Investigation Centre of Strasbourg University Hospital.

### Stimuli and apparatus

The stimulus was a red disc, size 1 degree of visual angle (dva), rotating along a white circular path (radius 2.75 dva), on a grey background. A fixation dot (size 1 dva) was presented at the centre of the screen during the trial.

Stimuli presentation and data acquisition was controlled by Matlab R2016b and Psychtoolbox-3^41^, running on a HP Zbook 17 with Windows 7. Stimuli were presented on a CRT screen with refresh rate 60 Hz, placed 60 cm from participants in a dimly lit room.

### Procedure

**Figure 1:**
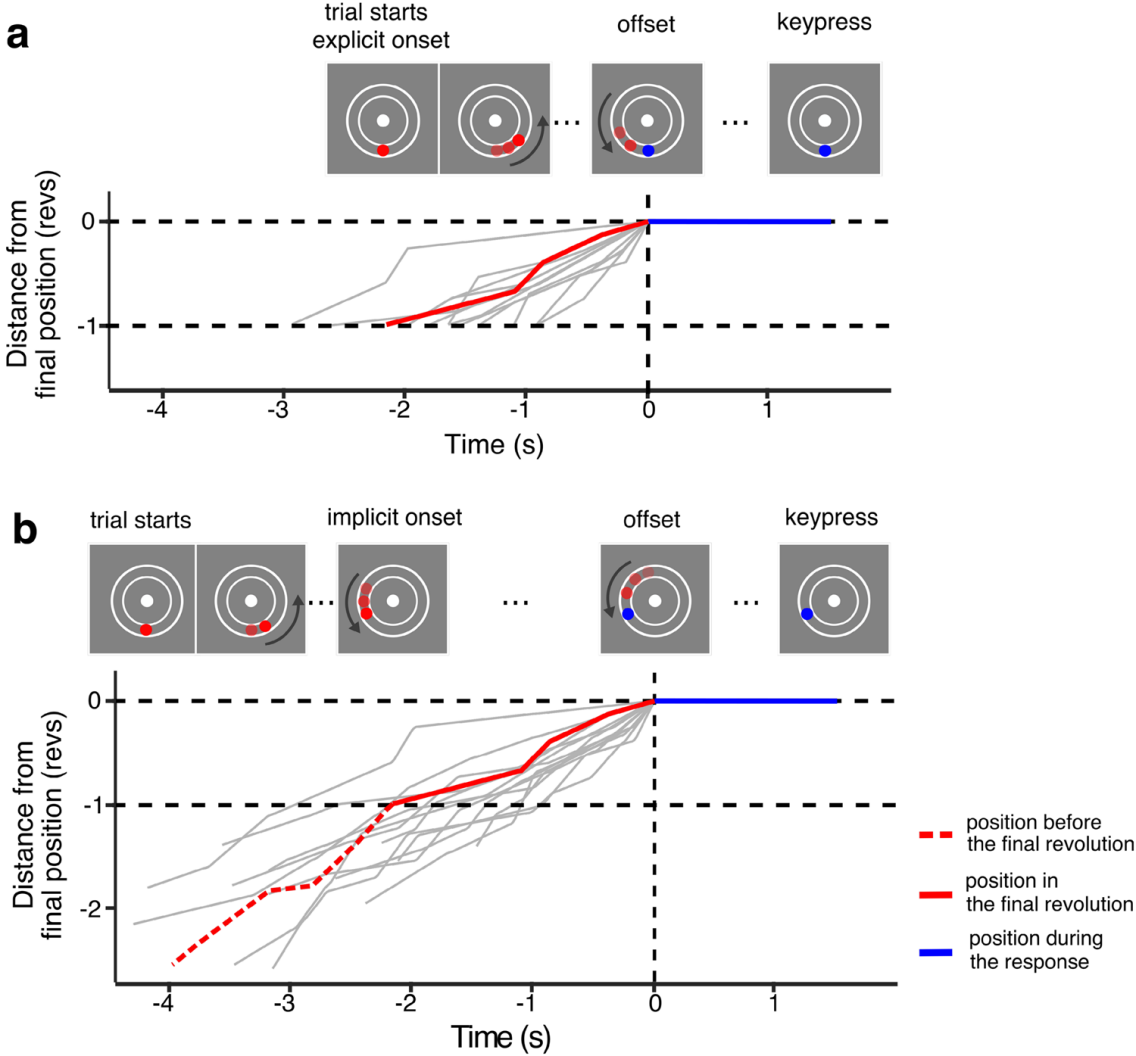
Illustration of the stimulus sequence in the explicit (**a**) and implicit (**b**) condition. (**a**) An example of changes in stimulus position as a function of time from the beginning of a trial in the explicit onset condition. The stimulus started moving in a counterclockwise direction at a constant speed. The stimulus made one revolution, and its speed could change during that revolution up to 5 times. Participants were asked to reproduce the duration of displacement, from the beginning to the end of the movement. The stimulus always started and stopped moving at the same position of the circular path (6 o’clock). (**b**) An illustration of the change in stimulus position as a function of time from the beginning of a trial in the implicit onset condition. As in the explicit onset condition, the stimulus always started moving from the same position in each trial, but its final position varied from trial to trial because it made more than one revolution. Participants did not know when or where the stimulus would stop, so they attended to the moving stimulus during the whole trial. The stimulus could change speed between 2 to 10 times during a trial but always moved at a constant speed between changes. Once the stimulus stopped, participants were asked to estimate how long it had been since the stimulus was in that same position and to reproduce that duration. The grey curves illustrate stimulus displacement in several different trials. The red curve shows one trial in which participants were asked to reproduce a duration of 2.3 s (duration corresponding to the spatial displacement from -1 to 0 on the y-axis). In both panels, position and time are aligned relative to the offset of the disc.

Each trial started with presentation of a central fixation disc, a circular path, and a static red disc located at bottom of the path. After 1 s, the red disc started moving around the path in a counter-clockwise direction, at a variable speed. After a variable duration, the disc stopped moving and changed colour to blue. Participants were asked to estimate the duration of the last full revolution, just before the stimulus stopped moving, and to provide their answer by then waiting the same duration before pressing a keyboard key. In other words, the task was to match the duration between the moment at which the disc stopped and the keypress, to the duration between the moment at which the disc stopped and the last time it had been at that same position in the circular path (i.e. at the beginning of the final revolution; Fig. 1). The total duration of the displacement varied from trial to trial, and differed according to the experimental condition. In the explicit onset condition, participants were informed the disc always made a single revolution, and their task was to estimate and reproduce the duration of the displacement, from the beginning to the end. In the implicit onset condition, the disc made more than one revolution, and participants were instructed to estimate and reproduce only the duration of the final revolution.

During the trial, the disc moved with varying speed (0.125–2.5 revolution per second), changing speed up to 10 times during the trial (up to 5 times in the final revolution). In both tasks, durations to be estimated were distributed log-normally, with median duration of 1.63 s (MAD = 0.6 s). In the implicit onset condition, median displacement duration before the final revolution (not including the revolution to be estimated) was 2.1 s (MAD = 1.3 s). The task and its validation are detailed elsewhere^27^.

On each trial, after participants had reproduced the estimated duration in the temporal task, they were asked to self-estimate their performance: whether the performance on that trial was better or worse than their average performance (single stimuli method, used to reduce the effects of idiosyncratic biases known to occur with rating scales e.g.^32,42^).

The two conditions were tested in separate blocks and task order was counterbalanced between participants. Participants completed 70 trials in each condition, preceded by 20 practice trials with feedback. The experiment lasted about 20 min.

### Drink contents

The BAL drink consisted of eight amino acids: Isoleucine (15 g), Leucine (22.5 g), Lysine (17.5 g), Phenylalanine (12.5 g), Threonine (10.0 g), Tryptophan (2.5 g), Tyrosine (12.5 g) and Valine (17.5 g), all dissolved in 400 ml of water. Because of its unpleasant taste and smell Methionine (5.0 g) was given in capsules. The APTD drink consisted of the same amino acid mixture, except for Tyrosine and Phenylalanine. The amount of each amino acid was reduced by 15% for the female participants, to account for differences in body weight.

### Data analysis

Data analysis was conducted in the R studio environment^43^. We used (generalised) linear mixed effect models since they allow random effects at the participant level, which models the variability of effects between participants (“lme4” and “car” packages,^44–46^). The significance of the factors was assessed by Wald’s Chi-square test method^47^). Prior to statistical analyses, we excluded trials on which the temporal error deviated more than 2 standard deviations from the mean temporal error (5% of the most extreme responses).

### Amino acids

Plasma concentrations of phenylalanine, tyrosine, and large neutral amino acids (LNAAs) were measured by liquid chromatography-mass spectrometry (Waters Xevo TQ-S micro). Brain availability of dopamine precursors can be approximated by the ratio of tyrosine plasma concentration to the sum of the concentration of the other LNAAs, since they compete for active transport across the blood–brain barrier^48,49^).

## Results

### DA precursor availability

To test whether our APTD manipulation was effective, we calculated the ratio of tyrosine to other large neutral amino acid plasma concentrations. At baseline, there was no difference in availability of DA precursors between the two drink conditions (t_baseline_(16) = 0.54, p = 0.6). Importantly, just before performing the behavioural tasks (~ 4 h later) the difference in plasma DA precursor availability was considerably smaller after the APTD drink compared to the BAL drink (t_test_(16) = -11, p < 0.001). These results have already been reported in another publication, together with findings of other timing tasks tested in the same protocol in a larger sample size^12^.

### Temporal estimation

**Figure 2:**
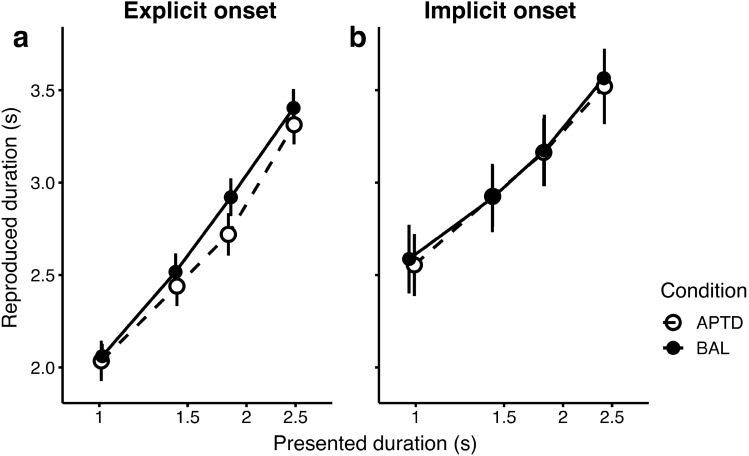
Performance in the temporal estimation task in the explicit (**a**) and implicit (**b**) onset temporal estimation tasks. For the purposes of visualisation, the presented duration is binned into four equally sized quantiles, and the reproduced duration is averaged for each of the bins. The BAL and APTD drink conditions are shown in filled and open symbols, respectively. Error bars indicate the standard error of the mean between participants.

To visualise performance in the temporal estimation task, we binned the presented duration into four approximately equally sized bins, and averaged the reproduced duration in each bin, separately for the two tasks and sessions. The reproduced duration varied in line with the presented duration in both temporal tasks (Fig. 2). To quantify effects we submitted the data to a linear mixed effect model, with reproduced duration as the dependent variable, and the logarithm of the presented duration (to account for the non-linearity of the perceived duration, mean-centered), task (implicit or explicit onset condition) and drink type (BAL or APTD) as the predictors. The random structure consisted of the random intercept and random slopes for the two fixed factors at the level of participant, to account for additional variability (the maximal random structure supported by the data). Note that the analysis was done at the single trial level, and that binning shown in Fig. 1 was done only for the purposes of visualisation. There was a significant effect of the logarithm of the presented duration (χ^2^(1) = 989.056, p < 0.001) and the temporal task type (χ^2^ (1) = 13.35, p < 0.001), as well as their interaction (χ^2^ (1) = 22.39, p < 0.01). The reproduced duration increased with the increase in the logarithm of presented duration (β = 3.187, SE = 0.101), indicating that participants performed the reproduction task as expected. We also found an interaction between the logarithm of presented duration and the temporal task (β = − 0.68, SE = 0.144), indicating that the slope was shallower for the implicit onset condition, as expected from previous work^27,50^. We also found a main effect of temporal task: on average, the reproduced duration was longer in the implicit onset task (β = 0.439, SE = 0.120). We also found large overestimation of perceived duration in both implicit and explicit conditions (the intercept of the model was 2.612 (SE = 0.106), whereas a lack of bias would have corresponded to an intercept of 1.63). The bias is consistent with previous work showing that the perceived duration of moving objects is overestimated compared to that of static objects^51,52^.

There was no significant main effect of drink type (χ^2^ (1) = 1.17, p = 0.27). Importantly, there were significant interactions between drink type and presented duration (χ^2^ (1) = 4.177, p < 0.05), and between drink type and temporal task (χ^2^ (1) = 6.463, p < 0.05).

The significant interaction between drink type and the presented duration (β = 0.293, SE = 0.143) suggests that the slope between the logarithm of presented duration and the reproduced duration became shallower in the APTD relative to BAL drink condition.

Furthermore, the interaction between the drink type and temporal task (β = − 0.079, SE = 0.03) indicates that the difference between the reproduced durations in the two tasks was smaller in the BAL than the APTD drink condition. Contrasts showed that in the BAL condition the difference between effects for the explicit relative to the implicit task was − 0.36 (SE = 0.139, z-ratio = − 3.654, p < 0.01), and in the APTD condition − 0.439 (SE = 0.120, z-ratio = − 3.654, p < 0.01).

Although a three-way interaction between task, drink type and the logarithm of the presented duration only tended towards significance (χ^2^ (1) = 3.766, p = 0.052), comparison of slopes for the two temporal tasks indicated a difference between slopes in the explicit, but not implicit, onset task (explicit: BAL – APTD, difference in effect = 0.2928 (SE = 0.143), z-ratio = 2.044, p < 0.05; implicit: BAL – APTD, difference in effect = − 0.0981 (SE = 0.141), z-ratio = − 0.694, p = 0.4877). The model explained a 0.68 proportion of variance in the data (conditional, marginal = 0.28^53^). A complementary analysis on absolute errors in reproduction shown in Supplementary Information further corroborates evidence for an interaction between the temporal task and drink condition on the performance.

### Confidence estimation

**Figure 3:**
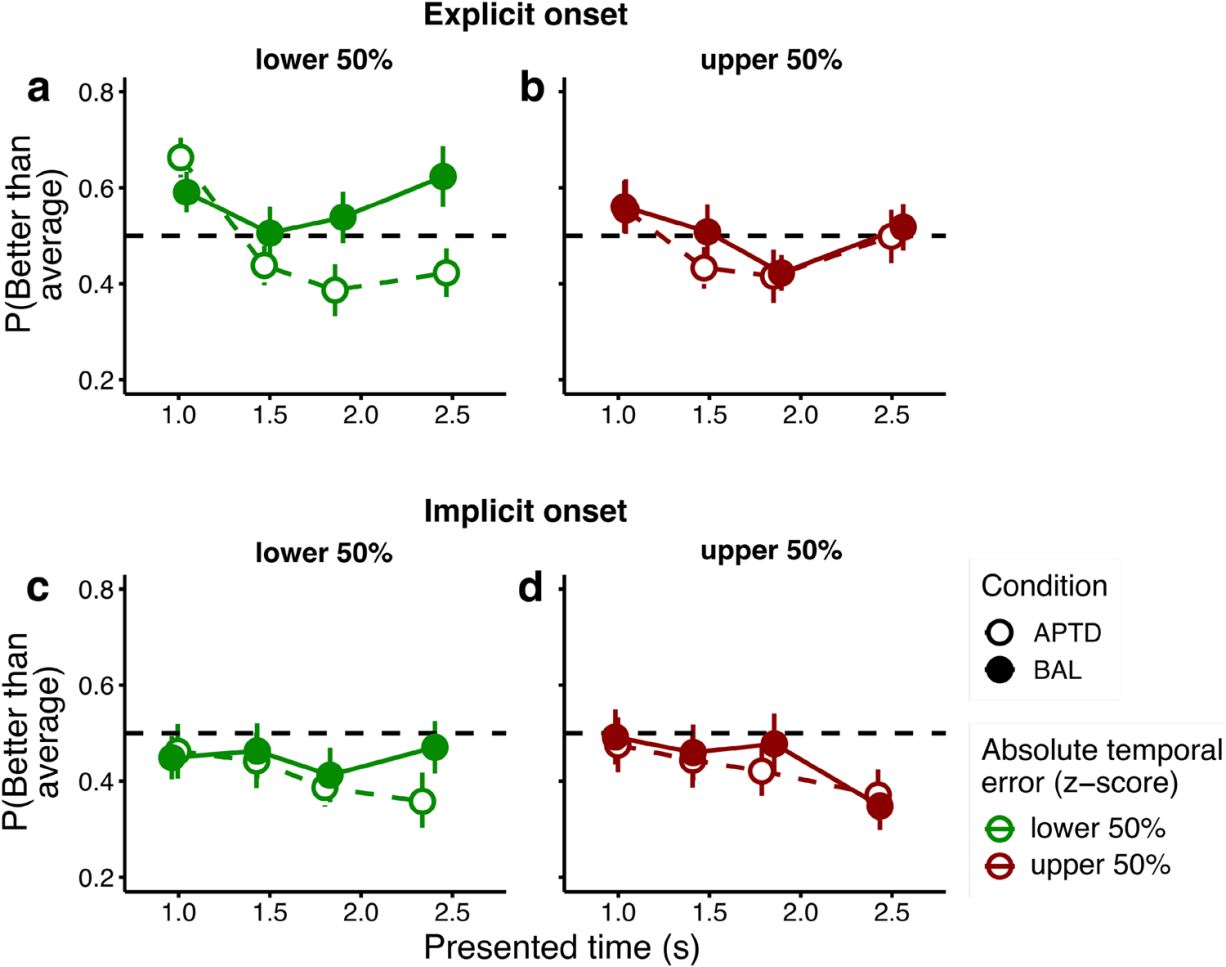
Results of the confidence judgement task. For the purposes of visualisation, we binned the presented duration into four equally sized bins and split the absolute standardised temporal error into two bins (smaller or greater than the median). The probability of rating performance as better than average is plotted against the presented duration quantiles, separately for the APTD and BAL conditions (empty and filled symbols, respectively) and absolute standardised temporal error categories (colour coded, green and red symbols show trials on which the absolute standardised error was smaller and greater than the median, respectively). (**a**) and (**b**) Probability of estimating performance as better than average as a function of presented duration and drink condition in the explicit onset task. (**a**) and (**b**) show confidence judgements for trials in which the absolute standardised temporal error was lower (**a**) or higher (**b**) than median. There was a non-linear negative relationship between the presented duration and confidence in timing performance. This relationship was stronger in the APTD condition, and for smaller absolute standardised temporal errors. Finally, APTD increased the interaction between presented duration and the absolute standardised temporal error. (**c**) and (**d**) Probability of estimating performance in a trial as better than average as a function of presented duration and drink condition in the implicit onset task. (**c**) and (**d**) show confidence judgements for trials in which the absolute standardised temporal error in the implicit onset task was lower (**c**) or higher (**d**) than median. The confidence in performance decreased with longer presented durations. Error bars indicate the standard error of the mean between participants.

We assumed that in order to estimate confidence in their performance observers relied on estimations of how their performance on a given trial deviated from their average performance. Since average performance approximates to the mean absolute temporal error, we calculated deviation from the mean absolute error on each trial to analyse confidence estimation (absolute difference between the reproduced and presented duration, z-scored for each observer and condition, and averaged across different presented durations). We tested the contribution of the presented duration on confidence judgements as a separate predictor in the model, since the uncertainty of temporal estimates increases with presented duration, allowing us to test effects of both the absolute error and the presented duration on the confidence decision in the same model^14^. The proportion of trials estimated to be more successful than average is plotted against presented duration (four equally sized bins) in Fig. 3, separately for z-scores greater or smaller than the median temporal error (median-split only for the purposes of visualisation). As shown in Fig. 3, there was a complex relationship between confidence, absolute error and presented duration, which differed for different tasks and drink conditions.

To quantify these effects, we tested a complex generalised linear mixed effects model (binomial family with a logit link function), which included the presented duration and absolute standardised temporal error distributions as continuous predictors, and the temporal task and the drink condition as fixed factors. The dependent variable was a binary confidence decision (better or worse than average performance). Since visual inspection revealed a non-linear relationship between the presented duration and confidence (Fig. 3), we modelled the effect of the presented duration by including both a first order and a quadratic term in the model. We found a complex relationship between the predictors, whose coefficients are shown in Table 1 (for simplicity only significant predictors are shown in the table, a full list is shown in Table S4, together with technical details on the analysis and data transformation, and a justification of the use of a quadratic term). Given the two three-way interactions, we conducted separate analyses for the two temporal tasks to facilitate interpretation.

**Table 1:** Significant predictors of the model fitted to confidence judgements.

Predictor	β (*SE*)	χ^2^(df = 1)
Presented duration (centered)	− 0.587 (0.111)	27.96, p < 0.01
Presented duration^2^ (centered)	0.310 (0.070)	19.306, p < 0.01
Temporal task	− 0.327 (0.148)	4.86, p < 0.05
Absolute standardised error × presented duration	0.233 (0.109)	4.66, p < 0.05
Presented duration × drink condition	0.537 (0.153)	12.23, p < 0.01
Absolute standardised error × presented duration × temporal task	− 0.318 (0.152)	4.315, p < 0.05
Absolute standardised error × presented duration × drink condition	− 0.355 (0.149)	5.715, p < 0.05

In the explicit onset task, participants used information about the to-be reproduced duration when estimating confidence (both linear: χ^2^ (1) = 29.6, p < 0.01, β = − 0.616, *SE* = 0.113, and quadratic terms were significant: χ^2^ (1) = 16.88, p < 0.01, β = 0.397, *SE* = 0.1). There was a non-linear, negative relationship between presented duration and confidence: the longer the presented duration, the lower the probability that performance on that trial was judged better than average (Fig. 3a and b). Furthermore, there was an interaction between drink type and presented duration (χ^2^ (1) = 12.217, p < 0.01, β = 0.539, *SE* = 0.154), indicating that the negative relationship between presented duration and confidence was stronger in the APTD drink condition. We also found an interaction between the absolute standardised error and presented duration (χ^2^ (1) = 4.78, p < 0.05, β = 0.238, *SE* = 0.110): the greater the absolute standardised error, the weaker the relationship between presented duration and confidence (Fig. 3a and b). In fact, failing to estimate duration, i.e. when there was a large absolute standardised error, may lead to confusion between the different durations and a loss of the influence of duration on confidence. Interestingly, there was also a three-way interaction between presented duration, drink condition and absolute standardised error (χ^2^ (1) = 6.27, p < 0.05, β = − 0.375, SE = 0.150), indicating that the relationship between confidence and presented duration was stronger after APTD mainly for low absolute standardised error trials, with confidence decreasing with presented duration more in the APTD than in the BAL group (see Fig. S4 in the Supplementary material for model predictions as a function of different values of the absolute standardised error). There was no significant main effect of the absolute standardised error (χ^2^ (1) = 0.415, p = 0.519), the drink type (χ^2^ (1) = 2.2, p = 0.137), and no interaction between the absolute standardised error and drink type (χ^2^ (1) = 1.763, p = 0.184). The model explains a 0.085 (0.032 marginal) proportion of variability in the data.

By contrast, in the implicit onset task, presented duration was the only significant predictor (linear term: χ^2^ (1) = 10.267, p < 0.01, β = − 0.333, *SE* = 0.117, quadratic term not significant: χ^2^ (1) = 3.51, p = 0.06). The longer the presented duration, the lower the probability that performance on a given trial was judged as better than average (Fig. 3c and d). There was no significant main effect of the drink type (χ^2^ (1) = 1.55, p = 0.212), nor interaction with the presented duration (χ^2^ (1) = 1.031, p = 0.310). Furthermore, there was no effect of the absolute standardised error (χ^2^ (1) = 0.017, p = 0.896), nor interaction between the absolute standardised error and the presented duration (χ^2^ (1) = 0.608, p = 0.435) or the drink type (χ^2^ (1) = 1.03, p = 0.309), suggesting that participants did not evaluate their performance based on temporal error in a given trial. The three-way interaction between the three predictors was also not significant (χ^2^ (1) = 0.358, p = 0.549). This model explained a 0.162 proportion of the variability (0.011 marginal).

## Discussion

We investigated the nature and specificity of the role of dopamine in the processing of temporal information. Dopamine precursor availability specifically affected timing of an event whose onset was explicitly identified in advance, confirming dopamine’s role in the prospective encoding of duration, as opposed to an effect on the ability to reliably process duration information in general. This finding was corroborated by results of the self-evaluation of temporal performance, which was affected by dopamine precursor availability in the explicit onset condition only, further suggesting that dopamine is likely to be important for the reliability of the prospective internal clock.

Effects of the dopamine manipulation in the explicit onset condition are in general agreement with previous work implicating dopamine in explicit duration processing^5,6,9,12,54–56^. Of note, in our paradigm participants both estimated and reproduced the presented duration in the same dopaminergic state. Had dopamine activity affected both the encoding and the reproduction of temporal intervals in the same way, no effects of the manipulation on the reproduced duration should have been observed. Our results are consistent with previous work^6,9^ indicating that APTD induced an underestimation of duration at encoding, leading to a shorter duration during reproduction: in other words, the dopamine manipulation affected processing of temporal information during the presentation of the duration and its encoding, rather than during the reproduction phase.

Altered dopamine metabolism could affect explicit interval timing in two ways: by affecting reliability (increasing uncertainty) or by biasing the encoding of the temporal interval (changing the “clock speed”). Duration estimates in the explicit onset task were more biased towards the mean of the duration distribution after APTD, reflected in the shallower slope between presented and reproduced duration. This can be interpreted as a consequence of a computational strategy which combines the contextual prior with current sensory information, where the effect of the prior increases with increased uncertainty^50,57^. This is consistent with previous findings in mice^5^, and evidence that dopamine manipulation affects the uncertainty of temporal estimates in humans^29,30,58^. In addition, the APTD manipulation decreases the accuracy of temporal judgements^6^ and the dopamine antagonist haloperidol increases thresholds of auditory duration discrimination^54,55^, consistent with a reduced reliability hypothesis. Of note, we also observed a bias to underestimate duration after APTD, in addition to the putative differences in the uncertainty of the estimates. An alternative explanation, suggested in the revision process, is that the APTD manipulation affected the efficiency of the mechanism encoding perceived units of time (i.e. the accumulator), which would lead to greater underestimation for longer durations. However, since we observed a difference between the two tasks in the effect of the APTD manipulation, the effect on the accumulation would be specific to the explicit task, suggesting at least partially different mechanisms.

Importantly, we did not find evidence that performance in the implicit onset task was affected by decreased dopamine precursor availability. This dissociation is generally consistent with previous work showing a distinction between explicit timing and various implicit forms of timing^22,59,60^ and, more specifically, with a dissociation in the effects of APTD on temporal reproduction but not temporal prediction^12^. In our study, timing in the implicit task cannot be termed “retrospective” since participants know in advance that they will have to produce timing judgements. Nonetheless, the mechanisms involved might be similar to those proposed in retrospective tasks, which have been related to retrieved memory about events, or the number of changes detected or retrieved^17,20,21,61–65^. In the implicit onset condition participants had to rely on a memory trace of the stimulus trajectory to be able to reproduce the duration of the last revolution of the moving disc, once its final position had been revealed. The lack of APTD effect in the implicit onset task therefore provides further evidence that APTD affects the prospective encoding of stimulus duration, rather than its retrieval^6,9,12^. Indeed, previous work showed no evidence for APTD effects on the production of temporal intervals, when participants were asked to reproduce a one second duration from long-term memory^9^.

Given the putative role of working memory in the implicit onset task, and a close link between dopamine and working memory^66–71^, the lack of evidence for the effects in the implicit onset condition might be unexpected. However, different components of working memory might differently depend on dopamine, and previous work found no evidence for APTD-induced effect on working-memory performance^72–79^.

Performance in the confidence task revealed several important insights. First, we found that participants exploited different cues to assess their performance (e.g.^80,81^). In both explicit and implicit onset tasks, confidence about performance was related to the presented duration; a valid strategy for the self-evaluation of performance since the variability of temporal estimates scales with presented duration (e.g.^13^). Furthermore, we found an interaction between the presented interval and the absolute standardised temporal error on confidence in the explicit onset task only, indicating that observers took their temporal error into account when making confidence judgements (although this relationship also depended on the drink condition).

Second, our manipulation of dopamine precursor availability did not lead to a generalised impairment in metacognitive judgements, since there was no effect of drink type on confidence in performance in general. Instead, reduced dopamine precursor availability had an effect on metacognitive judgements in the explicit onset temporal task only, by perturbing the relationship between the presented and perceived duration, and consequently, the relationship between the perceived duration, temporal error and confidence. These results are in agreement with the hypothesis that the dopamine manipulation affected the reliability of temporal encoding. Previous work in non-human primates and rodents reported that dopamine signalling was related to subjective stimulus intensity and estimates of the probability of a choice being correct (e.g.^34,35,82^), suggesting that dopamine affects metacognitive evaluation in general. One possible source of difference between the previous work and our findings could be the manner in which confidence was assessed: while we asked for explicit reports of confidence using a method more resistant to response biases, previous work was based on statistical confidence inferred from performance in decision-making tasks.

In summary, we found that depletion of dopamine precursor availability altered performance in a prospective duration estimation task, in agreement with previous work^5,6,9,12^. However, when participants did not know in advance when the duration to be evaluated would start (the implicit onset task), we found no evidence that decreased dopamine precursor availability affected temporal estimation. These results are further corroborated by effects on the self-evaluation of performance. We found no evidence that metacognitive judgements in general were affected by decreased dopamine precursor availability; the effects were mediated by altered duration processing in the explicit onset task only. Our findings indicate that performance in these two temporal estimation tasks relies at least partially on different mechanisms, suggesting a specific role of dopamine signaling in the encoding of prospective temporal intervals^6^, rather than processing of temporal information in general^12,68^.

### Supplementary Information

Supplementary Information.

## Data Availability

The data that support the findings of this study are available on request from the corresponding author.
