# Supplementary material for: Dopamine precursor depletion affects performance and confidence judgements when events are timed from an explicit, but not an implicit onset
Source: Sci Rep. 2023 Dec 11;13:21933. doi: 10.1038/s41598-023-47843-w (PMC10713647; doi:10.1038/s41598-023-47843-w)
Supplement: Supplementary file 1 — Supplementary Information. [file 41598_2023_47843_MOESM1_ESM.pdf]

Supplementary material

Dopamine precursor depletion affects performance and confidence judgements when events are timed from an explicit, but not an implicit onset

Ljubica Jovanovic[1,2]*, Morgane Chassignolle[3], Catherine Schmidt-Mutter[4], Guillaume Behr[1], Jennifer T. Coull[3] and Anne Giersch[1]

[1]Inserm 1114, Strasbourg University, University Hospital of Strasbourg, Centre for Psychiatry, France
[2]Laboratoire des Systèmes Perceptifs, École Normale Supérieure, PSL University & CNRS
[3]Laboratoire des Neurosciences Cognitives (LNC), Aix-Marseille University & CNRS, France
[4]CIC Inserm 1434, CHU Strasbourg, France

Results

Visual Analogue Scales

In order to assess changes in the subjective level of arousal, fatigue and mood, participants completed Visual Analogue Scales (VAS) at the beginning (before taking the drink) and at the end (after completing the tasks) of each experimental session. VAS consisted of sixteen pairs of adjectives: awake - sleepy, calm - excited, strong - weak, confused - clear-minded, skilful - clumsy, lethargic - energetic, satisfied - unsatisfied, worried - calm, slow - fast, tense - relaxed, attentive - sleepy, incompetent - competent, happy - sad, hostile - friendly, interested - bored and reserved - social. Ten cm lines were placed between each pair of adjectives and participants responded by choosing the location on the lines that best described their subjective estimate.

Paired t-tests did not reveal any evidence for significant effects of the condition (BAL or APTD) between answers on any of the items. These results were already published in [1], which reported results of different temporal tasks performed as a part of the same experimental protocol.

Temporal Estimation

Fitting two separate models for the explicit and implicit task confirmed that the effects of dopamine precursor manipulation were different for the two tasks (random structure consisted of random intercept and slope for drink type at the level of participant). In the explicit onset task, there was an effect of the logarithm of presented duration (χ^2 (1) = 1668.111, $p<0.01$, β = 3.2, *SE* = 0.079) and a significant interaction between the drink type and the logarithm of presented duration (χ^2 (1) = 5.70, $p<0.05$, β = 0.265, *SE* = 0.111), indicating a greater slope of the logarithm of presented duration in BAL than APTD drink type condition (Fig. 3a and S1a). The main effect of the drink type was not significant (χ^2 (1) = 1.84, p=0.175). The model explained 0.726 proportion of the variance (0.46 marginal). In contrast, in the implicit onset condition, the logarithm of the presented duration was the only

significant predictor (χ^2 (1) = 433.2187, p<0.01, β = 2.512, *SE* = 0.120), and there was no effect of the drink type (χ^2 (1) = 0.026, p = 0.871) or their interaction (χ^2(1) = 0.370, p = 0.543, Fig. 3b and S1b). The model explained 0.65 proportion of the variance (0.14 marginal).

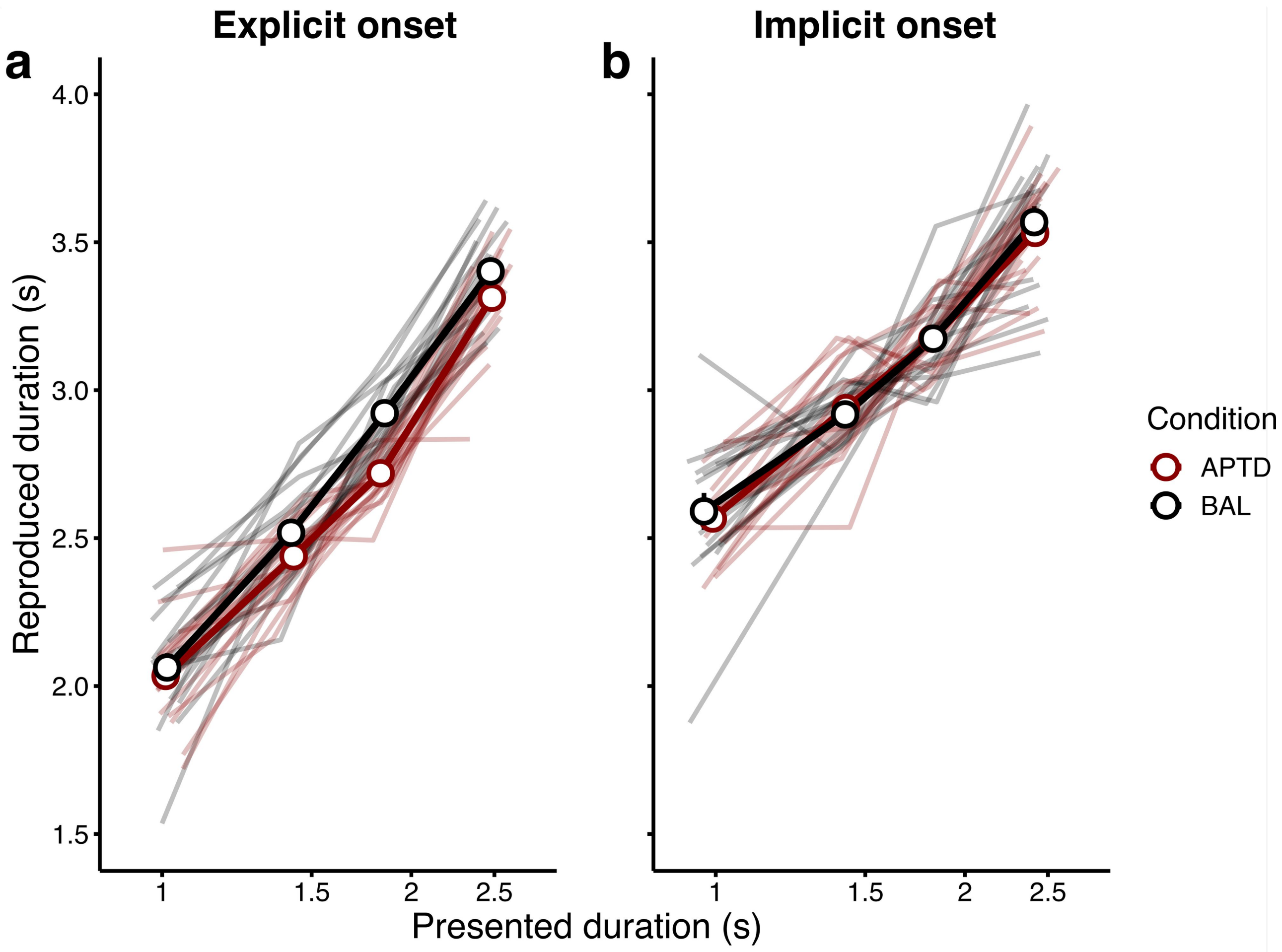

Figure S1. Performance in the temporal estimation task in the explicit (a) and implicit (b) onset temporal estimation tasks. For the purposes of visualisation, the presented duration is binned in four equally sized quantiles, and the reproduced duration is averaged for each of the bins. The BAL and APTD drink conditions are shown in black and red symbols, respectively. Performance of each participant is shown in thin lines (centred to individual mean reproduced duration) and average performance is shown in open symbols.

To further test effects of our manipulation, we also analysed absolute temporal errors (absolute difference between reproduced and presented duration, Fig. S2a) by means of a generalised linear mixed effect model (Gamma family distribution with a log link function). The temporal task and drink type were included as fixed effects, and dependent variable was absolute temporal error. The random structure consisted of both random intercepts

and slopes for temporal task and drink type at the level of participant. We found a significant main effect of the temporal task ($\chi^2(1) = 17.69$, $p<0.01$, $\beta = 0.383$, *SE* = 0.091), and an interaction between the temporal task and the drink type ($\chi^2(1) = 4.03$, $p<0.05$, $\beta = -0.100$, *SE* = 0.049). There was no significant effect of the drink type ($\chi^2(1) = 2.75$, $p=0.097$). The model explained 0.25 proportion of the variance (0.1 marginal). The significant interaction suggests that while the absolute error was different in the two temporal conditions (i.e. higher in the implicit onset task), the difference depended on the drink type, and was smaller in the BAL condition. Estimated marginal effects and their corresponding 95% confidence intervals are shown in Fig. S2b. (contrast for the two conditions (BAL – APTD) in the explicit onset task: 0.119 (0.072), z-ratio = 1.66, p = 0.097; contrast for the two conditions (BAL – APTD) in the implicit onset task: 0.0193 (0.0852), z-ratio = 0.226, p=0.821)

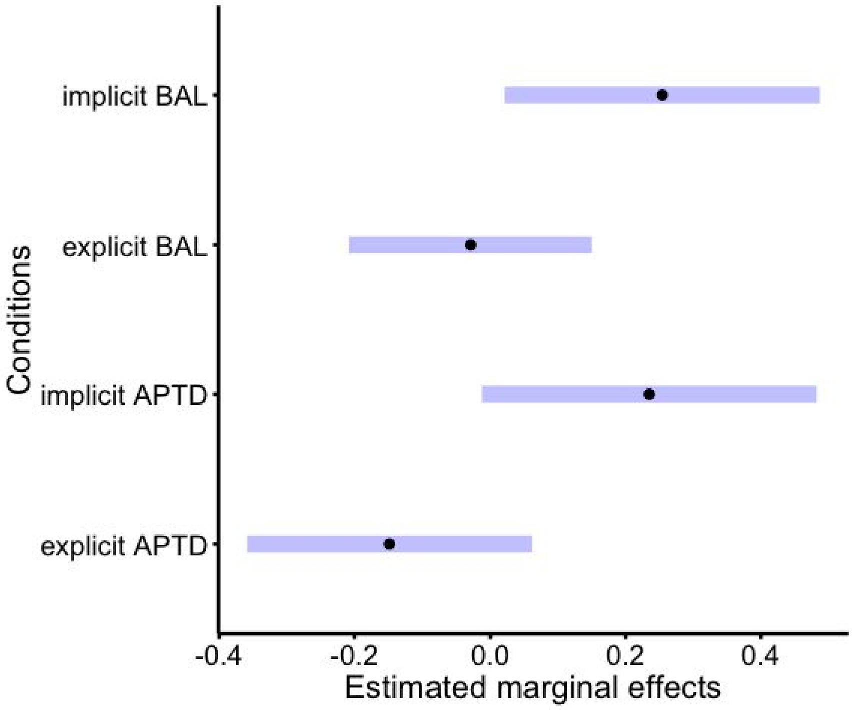

Figure S2. Estimated marginal effects with corresponding 95% confidence intervals.

Confidence estimation

To quantify effects on the self-evaluation of performance, we tested a complex generalised linear mixed effects model, which included the presented duration and absolute standardised temporal error distributions as continuous predictors, and the temporal task and the

drink condition as fixed factors. Since visual inspection revealed a non-linear relationship between the presented duration and confidence estimation, we modelled the effect of the presented duration by including both a first order and a quadratic term in the model (centred to reduce correlation in the interaction term, tested only as a main effect (no interactions with other predictors)). Therefore, the main outcome of this analysis and conclusions drawn from the analysis do not depend on the introduction of this predictor, but since including this predictor improves the fit of the model to the data ($\chi^2(1) = 19.536$, $p < 0.001$) we decided to include it in the model. The continuous predictors were standardised prior to the analysis, to reduce micro-multicollinearity in the model [2]. The random structure consisted of slopes for the temporal task and drink condition, and the intercept at the level of participant. We found a complex relationship between the predictors, and coefficients are shown in Table S1.

Table S1. Coefficients for the full model for the self-evaluation task

**P(Better than average) ~ Presented duration^2 + Presented duration x Absolute standardised error x Temporal task x Drink condition**		
Predictor	β (*SE*)	$\chi^2(1)$
Presented duration (centered)	-0.586 (0.110)	27.96, p < 0.01
Presented duration^2 (centered)	0.300 (0.0709)	19.306, p < 0.01
Absolute standardised error (z-score)	-0.039 (0.062)	0.397, p = 0.530
Temporal task	-0.327 (0.1488)	4.865, p < 0.05
Drink condition	0.258 (0.1748)	2.198, p < 0.138
Absolute standardised error x Presented duration	0.233 (0.108)	4.65, p < 0.05
Absolute standardised error x Temporal task	0.026 (0.0909)	0.085, p = 0.771
Presented duration x Temporal task	0.168 (0.1597)	1.112, p = 0.291
Absolute standardised error x Drink condition	-0.118 (0.090)	1.740, p = 0.187
Presented duration x Drink condition	0.537 (0.154)	12.229, p < 0.01
Temporal task x Drink condition	-0.086 (0.144)	0.357, p = 0.550
Absolute standardised error x Presented duration x Temporal task	-0.318 (0.151)	4.315, p < 0.05
Absolute standardised error x Presented duration x Drink condition	-0.351 (0.148)	5.715, p < 0.05
Absolute standardised error x Temporal task x Drink condition	0.026 (0.128)	0.044, p = 0.834

Presented duration x Temporal task x Drink condition	-0.351 (0.220)	2.521, p = 0.112
Absolute standardised error x Presented duration x Temporal task x Drink condition	0.249 (0.212)	1.366, p = 0.242

This model explained a relatively small proportion of the variance of confidence judgements, conditional $R^2 = 0.095$ (conditional; 0.03 marginal). Since this model was rather complex, we evaluated the multicollinearity of the predictors by means of the variance inflation factor (*vif*), which showed small and moderate variance inflation (between 1.2 and 4.2, all smaller than 5).

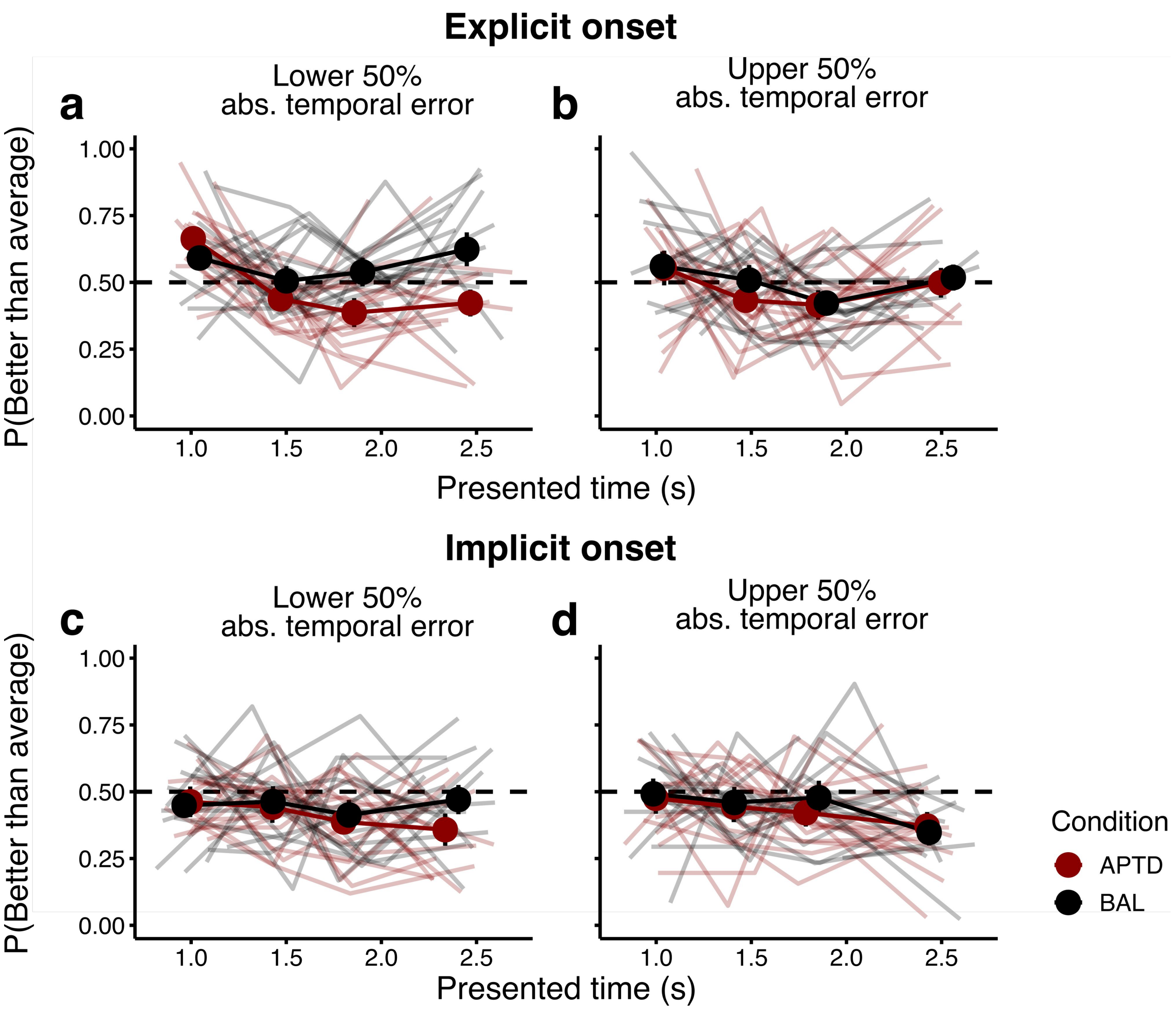

Figure S3. Individual results in the confidence estimation task. (a) and (b) Probability of estimating performance in a trial as better than average as a function of the presented duration and the drink condition in the

explicit onset task, shown separately for low (a) and high (b) absolute standardised temporal error. (c) and (d) Probability of estimating performance in a trial as better than average as a function of the presented duration and the drink condition in the implicit onset task, shown separately for low (c) and high (d) absolute standardised temporal error. Confidence in performance decreased with increased presented duration. Results from individual participants are shown with thin lines, and average performance is shown as circles.

Given the two three-way interactions, we conducted separate analyses for the two temporal tasks to facilitate interpretation (with random intercept and slope for the drink condition as random effects), as reported in the main manuscript. Outcome of the analysis for the explicit onset task is represented in Figure 4. Predicted probabilities of estimated performance in a trial as better than average are shown as a function of the presented duration (centered), for three values of the z-scored absolute standardised error. Predictions for the three values of the absolute standardised error are shown, in order to illustrate the three-way interaction between the presented duration, drink condition and the absolute standrdised error. As the absolute error increases (the performance deviates more from the average), the difference between the performance in the two conditions becomes more similar, and the effect of the presented duration less pronounced in the APTD condition.

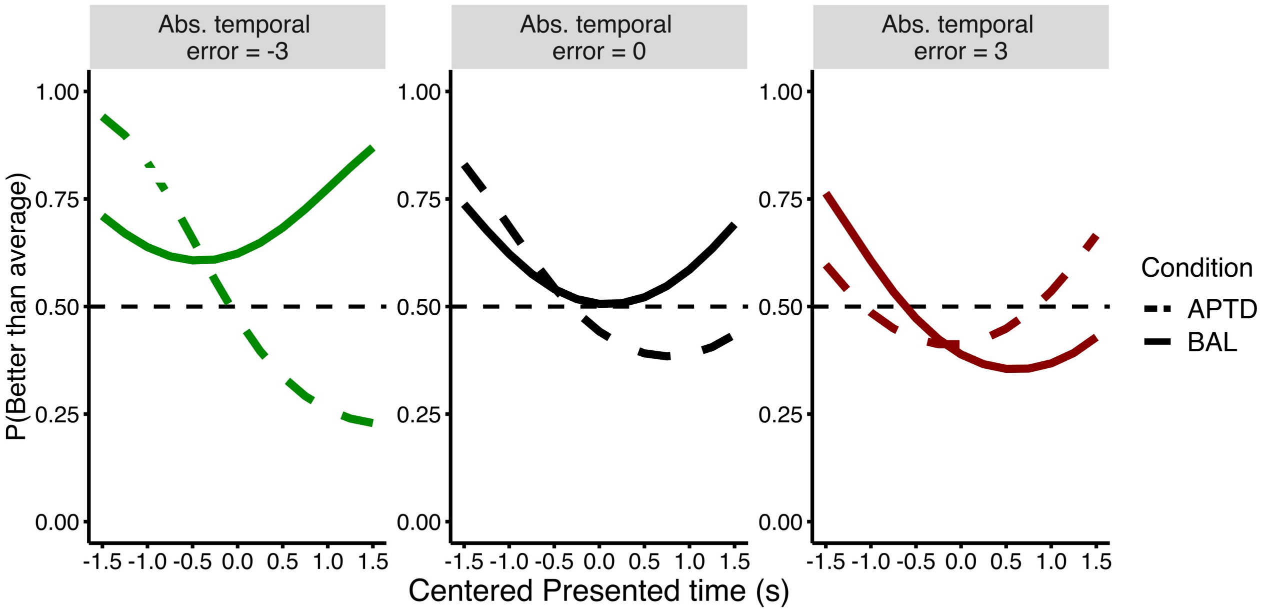

Figure S4. Predicted probabilities of estimated performance in a trial as better than average from the generalised linear model fitted to confidence responses in the explicit onset temporal condition, as a function of the presented duration (centered), for three values of the z-scored absolute standardised error. To illustrate the three-way interaction between the presented duration, drink condition and the absolute standardised error, we plotted predictions for the three values of the absolute standardised error. As the absolute error increases (the performance deviates more from the average), the difference between the performance in the two conditions becomes more similar, and the effect of the presented duration less pronounced.

References

[1] Chassignolle M., Jovanovic L., Schmidt-Mutter C., Behr G., Giersch A., Coull J. T. Dopamine precursor depletion in healthy volunteers impairs processing of duration but not temporal order. J Cogn Neurosci. **33**, 946-963 (2021).

[2] Iacobucci, D., Schneider, M. J., Popovich, D. L. & Bakamitsos, G. A. Mean centering helps alleviate “micro” but not “macro” multicollinearity. Behav Res Methods. **48**, 1308-1317 (2016).
